# PKA-Mediated Phosphorylation of SFRP4 Promotes Wnt/β-Catenin Activation and Cancer Stemness in Gastric Cancer

**DOI:** 10.3390/ijms26125572

**Published:** 2025-06-11

**Authors:** Yoo-Lim Jhe, Suji Lee, Youjin Jung, Jae-Ho Cheong

**Affiliations:** 1 Department of Medical Science, Graduate School, Yonsei University, Seoul 03722, Republic of Korea; yoolimj@yuhs.ac (Y.-L.J.); goamygo647@yuhs.ac (Y.J.); 2 Department of Surgery, Yonsei University College of Medicine, Seoul 03722, Republic of Korea; 3 Brain Korea 21 FOUR Project for Medical Science, Yonsei University College of Medicine, Seoul 03722, Republic of Korea; 4 Department of Biomedical Systems Informatics, Yonsei University College of Medicine, Seoul 03722, Republic of Korea; sujee425@naver.com

**Keywords:** SFRP4, PKA, cancer stemness, Wnt signaling, phosphorylation

## Abstract

Secreted Frizzled-related protein 4 (SFRP4) has been identified as a patient-level biomarker of the stem-like subtype of gastric cancer (GC), which is associated with poor prognosis and resistance to chemotherapy. Although multiple studies have documented the clinical significance of SFRP4 in GC, its mechanistic role in the stem-like subtype remains incompletely understood. In this study, we elucidate how phosphorylation of SFRP4 by protein kinase A (PKA) converts it into a Wnt signaling agonist. We began with a phosphoproteomic database search to identify candidate kinases that phosphorylate SFRP4. Co-immunoprecipitation assays revealed a direct interaction between PKA and SFRP4, and in vitro kinase assays confirmed that PKA phosphorylates SFRP4 at key threonine residues. Phosphorylated SFRP4 then associates with β-catenin, augmenting Wnt-driven transcriptional activity. Importantly, pharmacological inhibition of PKA significantly reduced SFRP4 phosphorylation and suppressed stemness-associated phenotypes, such as sphere formation, migratory capacity, and chemoresistance, in gastric cancer cells. Collectively, our data demonstrate that PKA-mediated phosphorylation of SFRP4 enhances cancer stemness-related properties in GC through Wnt signaling. Furthermore, these results highlight the PKA–SFRP4 axis as a promising therapeutic target in the stem-like subtype of GC.

## 1. Introduction

Gastric cancer (GC) ranks as the fifth most lethal malignancy worldwide [1], and its poor response to standard therapies is largely driven by pronounced intra-tumoral heterogeneity [2]. To guide precision treatment, multiple groups have applied molecular classification schemes—Singapore [3], The Cancer Genome Atlas (TCGA) [4], the Asian Cancer Research Group (ACRG) [5], and the Yonsei Cancer Center (YCC) [6]—revealing overlapping prognostic subtypes [7]. Notably, the Stem-like/EMT/Mesenchymal (SEM) subtype is characterized by genomic stability and marked chemoresistance [8,9], underscoring an urgent need to identify novel therapeutic targets for this high-risk patient population.

Aberrant activation of Wnt/β-catenin signaling is a key driver of GC aggressiveness [10]. Upon binding to Frizzled receptors and LRP5/6 co-receptors [11], Wnt ligands promote disassembly of the Axin destruction complex, liberating β-catenin to translocate into the nucleus and form a transcriptionally active complex with TCF4. This complex upregulates stemness-associated genes, such as *SOX2* [12], *POU5F1* [13], and *NANOG* [14], thereby enhancing metabolic plasticity [10], immune evasion [15], chemoresistance [16], invasion [17], and metastasis [18]. Tight regulation of this pathway is maintained by negative modulators, including Axin [19], secreted Frizzled-related proteins (SFRPs), and Dickkopf family members [20].

SFRP family proteins are traditionally viewed as extracellular Wnt antagonists, sequestering ligands or competing for Frizzled binding to inhibit pathway activation [21,22]. Paradoxically, SFRP4—classically, an antagonist [23,24,25]—also serves as a biomarker for the SEM subtype of GC [6], with elevated expression correlating with advanced T stage, EMT state, and poor clinical outcomes [26,27]. Of the five known SFRP isoforms, SFRP1, 2, and 5 are more associated with DNA sequences and Wnt targets than SFRP3 and SFRP4 [22]. At the level of protein sequence, SFRP1, SFRP2, and SFRP5 possess disulfide bonds at both the N- and C-termini, whereas SFRP3 and SFRP4 contain five disulfide bonds at the N-terminus and two threonine phosphorylation sites at the C-terminus. Notably, phosphorylated SFRP4 can bind to β-catenin and translocate to the nucleus, forming complexes with β-catenin and TCF4. These complexes exhibit relatively high transcriptional activity compared to the β-catenin/TCF4 complex alone. Although mass spectrometry studies have detected threonine phosphorylation sites in the C-terminal domain of SFRP4 and phosphorylated SFRP4 has been shown to bind β-catenin/TCF4 complexes [22], the upstream kinase and the precise intracellular mechanism remain undefined.

Protein kinase A (PKA) is a cAMP-activated serine/threonine kinase that regulates diverse cellular processes, including β-catenin stabilization via phosphorylation at S675 [28]. While dysregulated cAMP–PKA signaling contributes to tumorigenesis [29], a direct link between PKA and SFRP4 has not been established. Here, we demonstrate that PKA phosphorylates SFRP4 at threonine residues T186 and T189, promoting its association with β-catenin/TCF4 and thereby enhancing Wnt transcriptional activity. Moreover, pharmacological inhibition of PKA attenuates SFRP4-driven stemness phenotypes in GC cells. These findings elucidate a novel intracellular function of SFRP4 and propose the PKA–SFRP4 axis as a therapeutic target in the stem-like subtype of GC.

## 2. Results

### 2.1. Overexpression of SFRP4 Correlates with Poor Prognosis in Gastric Cancer

**Figure 1:**
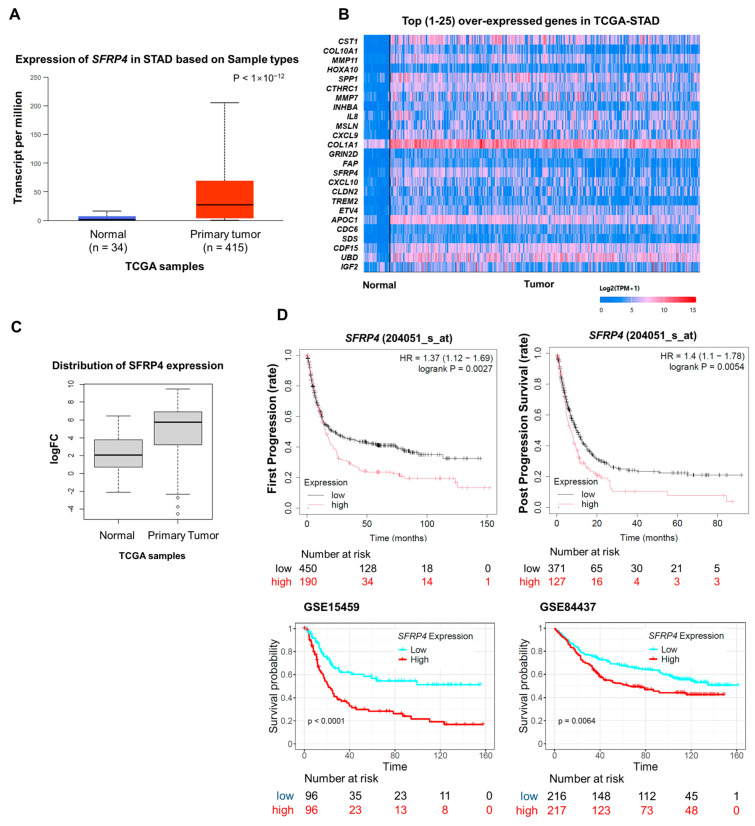
Clinical relevance of SFRP4 expression in gastric cancer. (**A**) UALCAN analysis illustrating the expression levels of SFRP4 mRNA in primary gastric tumors (n = 415) versus normal tissues (n = 34) using the TCGA database. (**B**) Top twenty-five genes overexpressed in stomach adenocarcinoma (STAD) in the UALCAN database. (**C**) The protein expression of SFRP4 in normal tissue versus primary tumors using TCGA-STAD samples. (**D**) Survival analysis with *SFRP4* high and low gastric tumors. First progression and post-progression survival rate upon *SFRP4* expression level (gene symbol: 204051_s_at) were generated using Kaplan–Meier plotter (upper) and overall survival rate in two distinct cohorts, GSE15459 and GSE84437 (lower). Abbreviations: STAD, stomach adenocarcinoma; TCGA, The Cancer Genome Atlas.

We have previously identified *SFRP4* as a patient-level biomarker of the stem-like subtype of GC, which is characterized by pronounced chemoresistance and unfavorable clinical outcomes [6]. To further evaluate its clinical relevance, we performed integrative analyses using the UALCAN portal and Kaplan–Meier Plotter databases. UALCAN analysis of TCGA data revealed that *SFRP4* is significantly upregulated in primary gastric tumors versus normal gastric mucosa (Figure 1A). Although *SFRP4* ranked among the most differentially expressed genes in stomach adenocarcinoma (STAD) through whole-transcriptome profiling, its expression varied widely across individual tumor samples (Figure 1B), suggesting context-dependent regulation. We further confirmed that the protein level of SFRP4 expression was significantly elevated in gastric tumor tissues compared with normal tissues (Figure 1C). Survival analyses across multiple independent cohorts showed that high *SFRP4* expression was consistently associated with decreased overall survival and disease-free survival (Figure 1D), concordant with our earlier observations in stem-like GC. Collectively, these data demonstrate that *SFRP4* is overexpressed in gastric tumors, exhibits intratumoral heterogeneity, and serves as an adverse prognostic indicator across diverse patient cohorts.

### 2.2. SFRP4 Induces Stemness-Related Properties in Gastric Cancer

**Figure 2:**
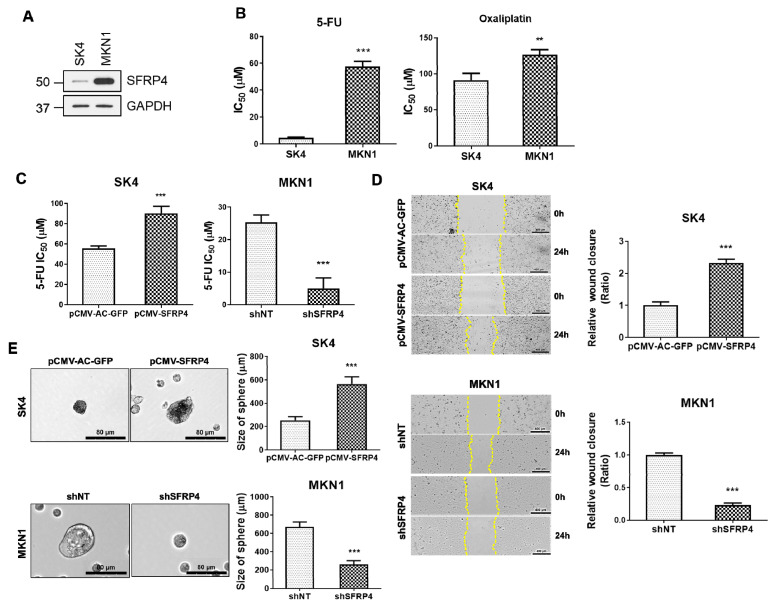
SFRP4 promotes stemness-related properties in gastric cancer. (**A**) Endogenous expression of SFRP4 was profiled in MKN1 and SK4 gastric cancer cell lines using Western blot. (**B**) The half maximal inhibitory concentration (IC_50_) values were evaluated following treatment with 5-fluorouracil (5-FU) and oxaliplatin for 72 h. (**C**) The IC_50_ value was evaluated following treatment of pCMV-SFRP4-SK4 cells and SFRP4-silenced MKN1 with 5-FU for 72 h. (**D**) Wound-healing ability was assessed following transfection of pCMV-SFRP4 into SK4 and shSFRP4 into MKN1 cells. Scale bars are 400 μm in inset images. Representative images were captured at 24 h of scratching. Yellow dashed lines indicate the wound’s edges. Cell motility was quantified using ImageJ (v1.52v) (**E**) Representative images of sphere-forming ability expressing pCMV-SFRP4 in SK4 and shSFRP4 in MKN1. Scale bars are 80 μm in inset images. The data of (**B**–**E**) were obtained through three independent experiments. The data are presented as the mean ± standard deviation (n = 3). *p*-values were assessed using a two-tailed Student’s *t*-test. ** *p* < 0.01, *** *p* < 0.001. Abbreviations: 5-FU, 5-fluorouracil; IC_50_, half maximal inhibitory concentration.

To investigate the functional role of SFRP4 in GC phenotypes, we first profiled endogenous SFRP4 protein levels and 5-fluorouracil (5-FU) sensitivity across a panel of GC cell lines (Figure S1A,B). Based on this profiling, cell lines were stratified according to SFRP4 expression levels and corresponding drug response data. Accordingly, SK4 cells (low SFRP4) and MKN1 cells (high SFRP4) were chosen as representative models of low and high stem-like phenotypes, respectively (Figure 2A). We next sought to determine whether endogenous SFRP4 expression is associated with chemoresistance in GC cells. Notably, SFRP4-high MKN1 cells exhibited elevated half maximal inhibitory concentration (IC_50_) values for both 5-FU and oxaliplatin. We then assessed stemness-associated traits—migratory capacity and tumor-initiating potential—by performing wound-healing and spheroid-formation assays following SFRP4 modulation (Figure S2A–C). Overexpression of SFRP4 in SK4 cells increased the IC_50_ of 5-FU and accelerated wound closure compared with vector controls (Figure 2C,D). These SFRP4-overexpressing SK4 cells also formed significantly more and larger spheroids, phenocopying the behavior of MKN1 cells (Figure 2E). In contrast, SFRP4 knockdown in MKN1 cells impaired wound healing, reduced spheroid formation efficiency (Figure 2D,E and Figure S2B), and lowered the IC_50_ of 5-FU (Figure 2C) compared with cells transduced with non-targeting shRNA controls. Together, these data demonstrate that SFRP4 promotes the acquisition and maintenance of stem-like properties and confers chemoresistance in GC cells.

### 2.3. SFRP4 Induces Stemness-Related Properties Dependent on Wnt Signaling

**Figure 3:**
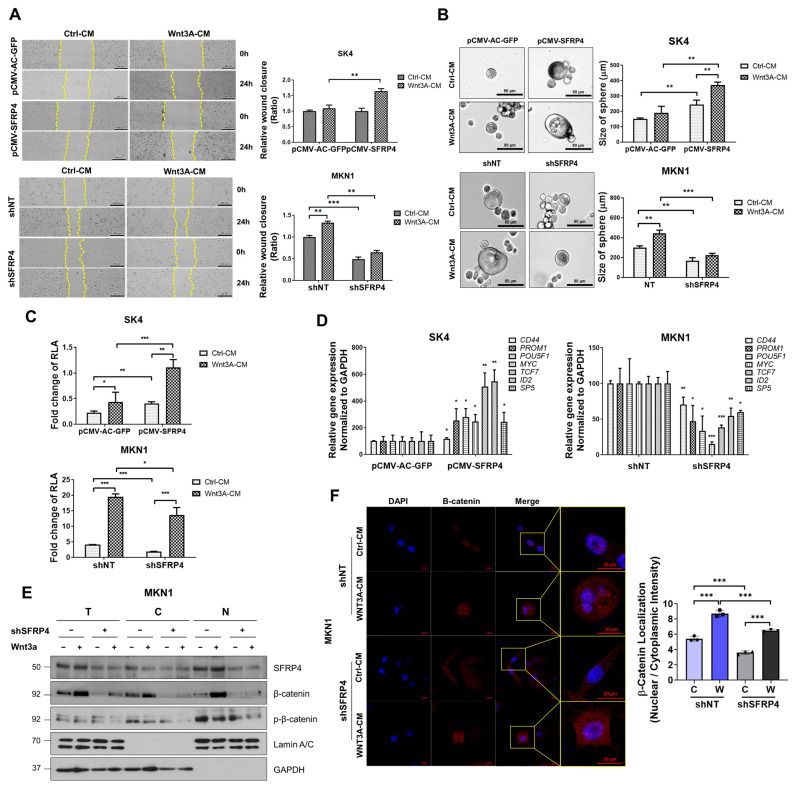
SFRP4 induces stemness-related properties dependent on Wnt signaling. (**A**) Wound-healing ability was assessed in SK4 and MKN1 cells transfected with the indicated constructs, treated with control-conditioned media (Ctrl-CM) and Wnt3A-conditioned media (Wnt3A-CM) for 16 h. Representative images of cell mobility were assessed following 24 h of scratching. Yellow dashed lines indicate the wound’s edges. Cell motility was quantified via ImageJ (v1.52). (**B**) To test SFRP4 function on cancer stemness, sphere formation assay was conducted in SK4 and MKN1 cells transfected with the indicated constructs. Scale bars are 80 μm in inset images. (**C**) Dual luciferase assay was performed to assess Wnt activity in pCMV-SFRP4 expressing SK4 and SFRP4 KD MKN1, treated with Ctrl-CM and Wnt3A-CM for 16 h. After being transfected with TopFlash/FopFlash plasmids for 72 h, relative luciferase activity (RLA) was evaluated. (**D**) Relative gene expression analysis was evaluated through qRT-PCR in SFRP4 expressing SK4 and SFRP4 knockdown MKN1. Expression levels of stemness-related and Wnt target genes were normalized to GAPDH. (**E**) Nuclear/cytosol fractionation was performed to assess the localization of both phosphorylated (p-β-catenin) and total β-catenin in MKN1 cells following SFRP4 knockdown and Wnt3A treatment. Cells were fractionated, and each fraction was immunoblotted with indicated antibodies. T, total; C, cytoplasmic; N, nuclear. (**F**) Immunofluorescence assay using confocal microscopy was performed to identify β-catenin localization. Control and SFRP4 KD MKN1 were treated with Wnt3A-CM for 16 h; DAPI (blue), β-catenin (red). Scale bars mean 20 μm. Nuclear and cytoplasmic β-catenin intensity was quantified using ImageJ (v1.52v), and the nuclear-to-cytosolic intensity ratio was plotted. C, Ctrl-CM; W, Wnt3A-CM (right panel). The data of (**A**–**D**,**F**) are presented as the mean ± standard deviation (n = 3), and *p*-values were assessed using a two-tailed Student’s *t*-test. * *p* < 0.05, ** *p* < 0.01, *** *p* < 0.001. Abbreviations: CM, conditioned media; p-β-catenin, phosphorylated β-catenin; RLA, relative luciferase activity.

Although SFRP4 is conventionally viewed as a Wnt antagonist, both our data and previous studies [22] indicate a context-dependent agonistic role in cancer. To determine whether SFRP4-driven malignant phenotypes rely on Wnt signaling, we modulated SFRP4 expression and treated cells with Wnt3A-conditioned medium (Wnt3A-CM), and then we assessed multiple stemness-associated traits and pathway activity. First, we assessed cell motility. Wound-healing assays revealed that SK4 cells overexpressing SFRP4 exhibited significantly enhanced migration compared with vector controls under both control-conditioned medium (Ctrl-CM) and Wnt3A-CM, whereas SFRP4 knockdown in MKN1 cells markedly impaired wound closure (Figure 3A). Consistent with these findings, sphere formation assays showed that SFRP4-overexpressing SK4 cells formed larger and more numerous spheres, while SFRP4-silenced MKN1 cells displayed reduced sphere-forming efficiency (Figure 3B). Next, we investigated whether SFRP4-induced stemness phenotypes were associated with changes in Wnt signaling activity. To quantify Wnt pathway activation, we performed a TCF/β catenin luciferase reporter assay. Overexpression of SFRP4 in SK4 cells significantly increased reporter activity, whereas SFRP4 knockdown in MKN1 cells led to a substantial decrease (Figure 3C). Quantitative RT-PCR analysis of canonical Wnt target genes (*CD44*, *POU5F1,* and *MYC*) corroborated these results. Expression of Wnt target genes, including *CD44*, *POU5F1*, and *MYC*, was elevated in SFRP4-overexpressing SK4 cells and reduced in SFRP4-deficient MKN1 cells (Figure 3D). To determine whether SFRP4 regulates Wnt signaling through modulation of β-catenin subcellular localization, we examined the distribution of β-catenin in nuclear and cytosolic compartments. SFRP4 knockdown markedly decreased nuclear β-catenin levels, an effect that was partially rescued by Wnt3A-CM treatment (Figure 3E). In addition, we assessed phosphor-β-catenin as an indicator of Wnt pathway activation. Notably, Wnt3A-CM treatment reduced nuclear localization of phosphor-β-catenin even under SFRP4 knockdown conditions, indicating that Wnt3A can partially compensate for the loss of SFRP4 (Figure 3E). Finally, immunofluorescence confirmed diminished nuclear β-catenin in SFRP4-knockdown MKN1 cells and restoration upon Wnt3A stimulation (Figure 3F). Taken together, these findings demonstrate that SFRP4 enhances Wnt/β-catenin transcriptional activity by promoting β-catenin nuclear translocation to drive stemness-related phenotypes in GC, in contrast to its canonical antagonistic function.

### 2.4. PKA Is a Putative Kinase for Phosphorylation of SFRP4

**Figure 4:**
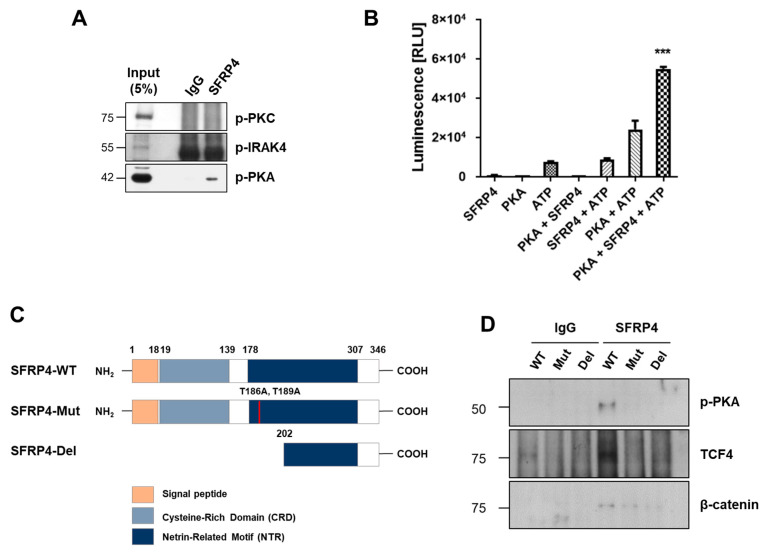
PKA phosphorylates SFRP4 and recognizes the C-terminal domain as a target site. (**A**) SFRP4 interacts with phosphorylated-PKA (p-PKA). Co-immunoprecipitation was performed using anti-SFRP4 antibody and total proteins extracted from MKN1 cell, followed by immunoblotting with anti-p-PKC, anti-p-IRAK4, and anti-p-PKA antibody, which were predicted as SFRP4 kinases. (**B**) An in vitro kinase assay showing phosphorylation of SFRP4 by PKA in the presence of ATP. Recombinant proteins were incubated under the indicated conditions, and kinase activity was measured through luminescence. The data present the mean ± standard deviation of three independent experiments. *** *p* < 0.001. (**C**) A scheme drawing of full-length wild-type (WT), mutation (Mut), and C-terminal deletion (Del) constructs of SFRP4. (**D**) Protein–protein interaction analysis of SFRP4 binding to interacting partners–p-PKA, TCF4 and β-catenin–following expression of WT, Mut, and Del constructs of SFRP4. Co-immunoprecipitation was performed using anti-SFRP4 antibody, followed by immunoblotting with the respective partner antibodies. Abbreviations: p-PKA, phosphorylated PKA.

Previous work has demonstrated that phosphorylated SFRP4 binds β-catenin and forms a complex with TCF4 to regulate Wnt target gene transcription [22]. To identify the kinase(s) responsible for phosphorylating SFRP4 at threonine residues T186 and T189, we conducted a phosphoproteomic database search. PKA emerged as the top candidate, with high scoring sites predicted using Quokka [30] (Tables S1 and S2). To validate these in silico predictions, we performed co-immunoprecipitation in MKN1 cells, which endogenously express high levels of SFRP4. Active, phosphorylated PKA (p-PKA) co-precipitated with SFRP4, whereas other predicted kinases (i.e., IRAK4 and PKC from GPS 5.0 [31] and the Human Protein Reference Database [32]) did not (Figure 4A). Next, in vitro kinase assays using purified PKA and recombinant SFRP4 showed significant ATP consumption, confirming direct phosphorylation (Figure 4B). To assess site specificity, we generated phosphorylation-deficient SFRP4 constructs: a double threonine-to-alanine mutant (T186A/T189A) and an N-terminal deletion (Figure 4C). Wild-type SFRP4 maintained robust interactions with p-PKA, TCF4, and β-catenin. In contrast, both double threonine-to-alanine mutant (T186A/T189A) and N-terminal deletion variants exhibited markedly reduced binding to p-PKA and TCF4, accompanied by impaired β-catenin association (Figure 4D). Reduced β-catenin binding was consistently observed across both mutant and deletion forms, suggesting that phosphorylation of SFRP4 at threonine residues facilitates the formation of the SFRP4–β-catenin–TCF4 complex. Collectively, these data demonstrate that PKA-mediated phosphorylation of SFRP4 at T186 and T189 is essential for stable assembly of the SFRP4–β-catenin–TCF4 transcriptional complex, underscoring a critical regulatory role for the PKA–SFRP4 axis in Wnt signaling.

### 2.5. Inhibition of PKA Decreases Wnt Signaling-Dependent Stemness in Gastric Cancer

**Figure 5:**
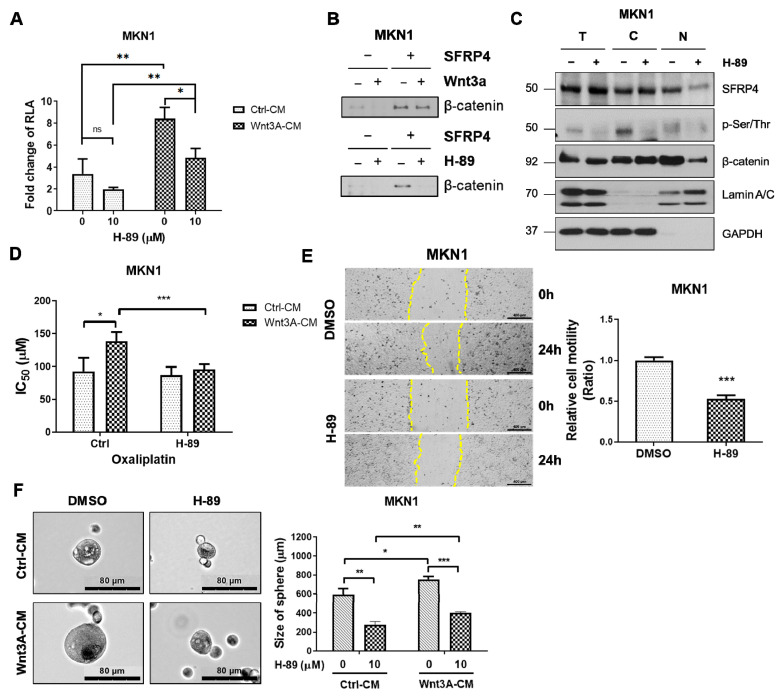
Inhibition of PKA decreases Wnt signaling-dependent stemness in gastric cancer. (**A**) Wnt luciferase activity was evaluated following treatment with 10 μM H−89 and Wnt3A-conditioned media (Wnt3A-CM) for 24 and 16 h, respectively. (**B**) Co-immunoprecipitation was performed following treatment with 10 μM H-89 and Wnt3a-CM for 24 and 16 h, respectively. (**C**) MKN1 cells were treated with the PKA inhibitor H-89, followed by nuclear/cytosolic fractionation. Each fraction was subjected to immunoblotting with the indicated antibodies to assess subcellular distribution. T, total; C, cytoplasmic; N, nuclear. (**D**) The IC_50_ value was evaluated following treatment with oxaliplatin for 72 h. (**E**) Wound-healing ability was evaluated following treatment with 10 μM H-89 for 24 h. Cell motility was captured following 24 h of scratching and evaluated via ImageJ (v1.52v). Yellow dashed lines indicate the wound’s edges. (**F**) Sphere-forming ability was evaluated by the size of the sphere. The data presented in (**A**,**D**–**F**) represent the mean ± standard deviation of three independent experiments. * *p* < 0.05, ** *p* < 0.01, *** *p* < 0.001. Abbreviations: CM, conditioned media; p-Ser/Thr; phosphorylated serine/threonine; RLA, relative luciferase activity.

To assess the functional importance of PKA in SFRP4-mediated Wnt signaling, we treated GC cells with the PKA inhibitor H-89 dihydrochloride [33]. H-89 markedly reduced global phosphorylated serine/threonine levels (p-Ser/Thr) (Figure S3A) and significantly suppressed Wnt-driven luciferase reporter activity, indicating that PKA activity is required to sustain SFRP4-dependent pathway activation (Figure 5A). Co-immunoprecipitation assays revealed that H-89 impaired the SFRP4–β-catenin interaction (Figure 5B), and nuclear/cytoplasmic fractionation showed a pronounced decrease in nuclear SFRP4 upon PKA inhibition, while total SFRP4 and β-catenin protein levels remained unchanged (Figure 5C and Figure S3B), indicating that PKA regulates subcellular localization of SFRP4 without altering its overall expression. To further assess phosphorylation-dependent nuclear localization of SFRP4, we employed a pan-p-Ser/Thr antibody due to the lack of commercially available phosphorylated SFRP4-specific antibodies. Upon H-89 treatment, p-Ser/Thr levels in the nuclear fraction were substantially reduced, suggesting diminished nuclear localization of phosphorylated SFRP4 under PKA inhibition. Functionally, depletion of SFRP4 in MKN1 cells lowered the IC_50_ for oxaliplatin even under Wnt3A stimulation, demonstrating that SFRP4 is critical for Wnt-driven chemoresistance (Figure 5D). Moreover, H-89 treatment significantly reduced sphere-forming efficiency (Figure 5E,F), confirming that PKA-dependent phosphorylation of SFRP4 is essential for its nuclear localization, β-catenin binding, and the acquisition of stemness traits in gastric cancer.

## 3. Discussion

Although SFRP4 is traditionally classified as a Wnt antagonist, emerging evidence underscores its context-dependent and even paradoxical roles in cancer. Aberrant Wnt signaling is a hallmark of numerous malignancies, including colorectal, hepatocellular carcinoma (HCC), breast, and GCs, where it drives proliferation, stemness, and resistance to therapy [34]. In line with this, clinical studies have linked high SFRP4 expression to favorable outcomes in breast, ovarian, and liver cancers, yet in the stem-like subtype of GC, it portends markedly worse prognosis [35]. Such divergent clinical correlations may reflect SFRP4’s modular structure, with two distinct domains that differentially engage Wnt ligands, receptors, or intracellular partners.

**Figure 6:**
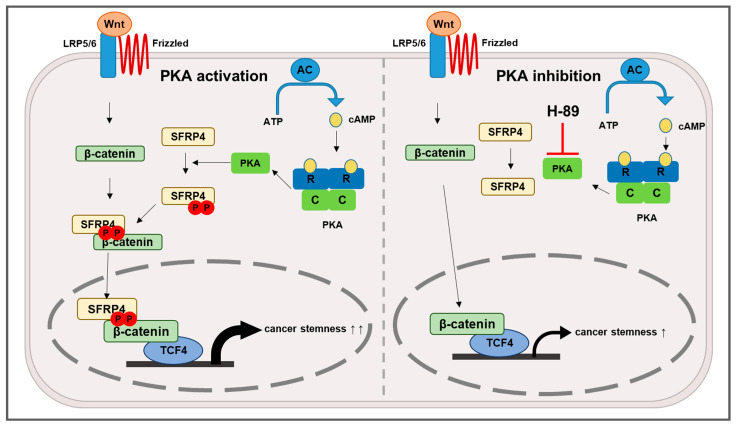
A proposed mechanism for the effect of PKA-mediated regulation of SFRP4 phosphorylation on GC stemness. Upon PKA activation, ATP is converted to cAMP, which then activates PKA by binding to its regulatory subunits, releasing the catalytic subunits. These catalytic subunits phosphorylate SFRP4 at specific threonine residues. Phosphorylated SFRP4 subsequently binds to β-catenin, facilitating the formation of a complex with TCF4. This SFRP4–β-catenin–TCF4 complex translocates to the nucleus. Within the nucleus, the complex activates the transcription of Wnt target genes. This process enhances cancer stemness properties, including increased proliferation, migration, and tumorigenicity. Symbols: black solid arrow, activation; red bar, inhibition; double-headed arrow, strong increase; single-headed arrow, moderate increase.

In this work, we delineate a mechanistic basis for SFRP4’s oncogenic function in GC by demonstrating that PKA-mediated phosphorylation converts SFRP4 into a Wnt agonist. Guided by prior observations that SFRP4 phosphorylated at T186 and T189 binds β-catenin/TCF4 to drive Wnt target gene transcription [22], we used phosphoproteomic prediction and in vitro kinase assays to identify PKA as the responsible kinase. Mutational and deletional analyses of SFRP4 confirmed that phosphorylation at these threonine residues is indispensable for its interaction with PKA, β-catenin, and TCF4. Pharmacologic inhibition of PKA abrogated Wnt transcriptional activity and attenuated stemness-associated phenotypes—including spheroid formation, migratory capacity, and chemoresistance—thereby establishing the PKA-dependent SFRP4 axis as a critical intracellular mechanism of Wnt hyperactive GC stemness (Figure 6).

Interestingly, β-catenin itself is a direct PKA substrate [28], suggesting that PKA may modulate multiple Wnt pathway components in coordination. Thus, PKA may simultaneously modulate multiple nodes within the Wnt signaling cascade. Future studies should elucidate how such coordinated phosphorylation events affect Wnt transcriptional output and stemness maintenance across distinct tumor contexts. While a phosphorylation site-specific SFRP4 is not currently available, we employed a pan-p-Ser/Thr antibody to indirectly assess SFRP4 phosphorylation. Given that the PKA-targeted residues (T186 and T189) are threonine residues, MKN1 cells were treated with the PKA inhibitor H-89 and subjected to subcellular fractionation, followed by Western blotting. A marked decrease in the pan-p-Ser/Thr signal was observed in all cellular compartments upon H-89 treatment, suggesting reduced phosphorylation of nuclear SFRP4. Although indirect, this result supports the idea that PKA-mediated phosphorylation enhances the nuclear localization of SFRP4, consistent with its role in stabilizing the nuclear β-catenin/TCF4 transcriptional complex. These findings complement our luciferase reporter assays and β-catenin localization data and reinforce the functional importance of SFRP4 phosphorylation in Wnt pathway activation.

Although PKA inhibitors, such as H-89, PKI, and KT5720, are widely used in cancer research, their limited specificity and the essential roles of PKA in normal physiology complicate therapeutic translation [36]. Moreover, PKA exhibits both tumor-promoting and tumor-suppressive functions depending on cellular context. These considerations support more selective strategies, such as disrupting the PKA–SFRP4 interface or developing agents that specifically block SFRP4 phosphorylation, rather than broad PKA inhibition.

In this context, it is also relevant to consider extracellular Wnt inhibitors, such as Dickkopf-1 (DKK1), which suppress Wnt signaling by preventing Wnt ligand binding to the LRP6 receptors [37]. While DKK1 functions upstream at the membrane level, our study reveals that phosphorylated SFRP4 amplifies Wnt signaling downstream through intracellular stabilization of the β-catenin/TCF4 transcriptional complex. Preclinical studies have demonstrated that DKK1-targeting agents, such as the monoclonal antibody DKN-01, exhibit promising antitumor effects in Wnt-driven malignancies, including esophageal, intrahepatic cholangiocarcinoma, and gallbladder cancer [38]. Given that DKK1 and SFRP4 act at distinct regulatory levels within the Wnt cascade, a dual-targeted approach combining extracellular blockade via DKK1 or its mimetics with intracellular inhibition of the PKA–SFRP4 axis may help overcome resistance mechanisms and produce more durable therapeutic responses. Such strategies could be especially beneficial in the stem-like subtype GC, which exhibits persistent Wnt activity through both canonical and noncanonical mechanisms. Moreover, as DKK1 inhibits Wnt ligand binding to LRP6, it may influence upstream regulation of β-catenin phosphorylation, particularly at the N-terminal Ser/Thr residues targeted by GSK3β. Suppressing LRP6-mediated signaling could enhance β-catenin degradation by promoting its phosphorylation at Ser33/37/Thr41, thereby antagonizing the downstream stabilizing effects mediated by phosphorylated SFRP4. Thus, DKK1 may act synergistically with intracellular inhibitors to modulate both the stability and nuclear function of β-catenin. Furthermore, to support this model, we experimentally confirmed that phosphorylation of β-catenin is reduced in the nucleus upon Wnt activation. Western blot analysis demonstrated a decrease in nuclear p-β-catenin (at Ser33/37/Thr41) following Wnt3A treatment, consistent with pathway activation and stabilization of the transcriptionally active β-catenin pool.

In addition, given the central role of LRP6 in transducing Wnt signals, recent studies have identified natural compounds, such as γ-tocotrienol, a vitamin E isoform, as potent inhibitors of LRP6-mediated Wnt/β-catenin signaling. γ-tocotrienol has shown promise in suppressing tumor growth and stemness by attenuating LRP6 activation [39]. Although not directly assessed in our study, future research may explore whether γ-tocotrienol can inhibit the function of SFRP4, especially under PKA-driven conditions. Mechanistic validation could involve treating GC cells with γ-tocotrienol in the presence or absence of Wnt3A and PKA to assess changes in β-catenin/SFRP4 nuclear translocation. Such investigations could expand the therapeutic potential of natural Wnt modulators in targeting the PKA–SFRP4–LRP6 axis in cancer stemness.

In summary, our data reveal a previously unrecognized oncogenic axis in GC, as PKA-dependent phosphorylation of SFRP4 switches it from a Wnt antagonist to a potent agonist, thereby driving stemness and chemoresistance. By demonstrating that PKA inhibition reverses these malignant phenotypes, we nominate the PKA–SFRP4 module as a promising therapeutic target for the refractory, stem-like subtype of GC. These findings lay the groundwork for the development of next-generation inhibitors that selectively abrogate SFRP4’s pro-tumorigenic activity without compromising global cAMP/PKA signaling, and its combined inhibition with extracellular antagonists, such as DKK1, warrants further preclinical investigation.

## 4. Materials and Methods

### 4.1. Cell Cultures, Reagents, and Antibodies

The human gastric cancer cell lines were obtained from the Korean Cell Line Bank (Seoul, Republic of Korea), cultured in RPMI-1640 medium (Hyclone, Logan, UT, USA) supplemented with 10% fetal bovine serum (Hyclone), 100 μg/mL penicillin/streptomycin (GenDEPOT, Baker, TX, USA), and 1 mM sodium pyruvate (Gibco, Waltham, MA, USA), and incubated in a 5% CO_2_-containing incubator at 37 °C. To establish stable SFRP4 overexpression or knockdown cell lines, retroviral SFRP4 ORF vectors and lentiviral shSFRP4 vectors (Origene, Rockville, MD, USA) were transformed into DH5-alpha competent cells, and positive transformants were selected on agar plates containing the appropriate antibiotic (100 μg/mL ampicillin or 15 μg/mL chloramphenicol). SFRP4-site directed mutant (T186A, T189A) expressing plasmid was purchased from Bioneer (Daejeon, Republic of Korea), and SFRP4 deletion form expressing plasmid was purchased from the Korea Human Gene Bank (Daejeon, Republic of Korea). A total of 1 × 10^6^ cells were transfected via electroporation (Nepa Gene, Chiba, Japan). The transfected cells were selected in RPMI-1640 with the appropriate antibiotic (1 mg/mL kanamycin or 0.5 μg/mL puromycin), and protein expression was validated via Western blotting. β-catenin (ab16051), p-PKA (ab75991), p-Ser/Thr (ab17464), and SFRP4 (ab154167) were from Abcam (Waltham, MA, USA); β-catenin (sc-7963) was from Santa Cruz Biotechnology (Dallas, TX, USA); p-β-catenin (Ser33/37/Thr41) (9561S), Lamin A/C (2032S), p-IRAK4 (11927S), and TCF4 (2569S) were from Cell Signaling Technology (Danvers, MA, USA); SFRP4 (LS-C314315) was from LifeSpan Biosciences, Seattle, WA, USA); GAPDH (G9545) was from Sigma-Aldrich (Milwaukee, WI, USA); p-PKC (06-822) and normal mouse IgG (12371) were from Millipore (Burlington, MA, USA); and Vinculin (700062) and secondary antibodies for rabbit (G21234), mouse (G21040), and Alexa Fluor 568 (A-11004) were purchased from Invitrogen (Thermo Fisher Scientific, Waltham, MA, USA.

### 4.2. Western Blotting

RIPA Lysis Buffer (Pierce, Rockford, IL, USA), supplemented with protease inhibitor cocktail and PMSF, was used for cell lysis and fractionation, and the resulting protein lysates were quantified via BSA assay. Equal amounts of protein were separated via SDS-PAGE and transferred to a PVDF membrane. After 1 h of blocking with 5% skim milk, the membranes were incubated overnight with primary antibodies at 4 °C. On the following day, the membranes were washed five times with TBS-T and further incubated with the appropriate secondary antibodies.

### 4.3. Wnt Luciferase Activity Assay

Cells were seeded into a 96-well white plate and, on the following day, transfected with 0.1 μg/well TopFlash/FopFlash plasmids purchased from Addgene (Watertown, MA, USA) using the TransIT-X2 Dynamic Delivery System (Mirus Bio, Madison, WI, USA) with Renilla luciferase plasmid as a control. Three days after transfection, firefly and Renilla luciferase activities were evaluated via the dual-luciferase reporter assay protocol (Promega Corporation, Madison, WI, USA).

### 4.4. Co-Immunoprecipitation

Cells were lysed in IP lysis buffer (Pierce) supplemented with protease inhibitor cocktail and PMSF. Equal amounts of protein lysates were incubated with specific antibodies for 2 h at room temperature, followed by the addition of an appropriate amount of magnetic beads and overnight rotation at 4 °C. The incubated samples were washed five times with PBS-T (PBS with 0.1% Tween), followed by boiling at 98 °C for elution, and magnetized samples were loaded on SDS-PAGE.

### 4.5. In Vitro Kinase Assay

In vitro kinase activity was evaluated via ADP-Glo Kinase Assay (Promega Corporation). ATP-to-ADP conversion curves were constructed to generate a standard curve using the manufacturer’s protocol. The kinase reaction was performed at room temperature for 30 min. After the kinase reaction, the remaining ATP was depleted by adding the ADP-Glo reagent. ADP produced by the kinase reaction was converted to ATP by adding Kinase Detection Reagent and detected via luminescence.

### 4.6. Nuclear/Cytoplasmic Fractionation

Cells were dissociated using trypsin, and washed with PBS following treatment with 10 μM H-89 for 24 h (Selleck Chemicals, Houston, TX, USA). Cell lysates were fractionated using a Nuclear/Cytoplasmic fractionation kit (Abcam). Cytoplasmic protein expression was confirmed with GAPDH, and nuclear protein expression was confirmed with Lamin A/C.

### 4.7. Sphere-Forming Assay

A total of 1 × 10^5^ cells/mL were seeded into 6-well ultra-low attachment adhesion plates (Corning, Lowell, MA, USA) with DMEM/F12 (Gibco) containing 5% FBS supplemented with 10 ng/mL insulin and 1 μg/mL hydrocortisone (Gibco). At every two-day interval, 500 μL of the medium was added per well. After 7 days of incubation, the overall sizes of the spheres of aggregates of cells were analyzed using photographical images. Representative images of spheres were captured via microscope at 200× magnification.

### 4.8. Wound-Healing Assay

Cells were seeded into 6-well plates and incubated until >90% confluence was reached. Straight wound lines were created by scratching the cells with a sterile 1000 μL pipette tip, followed by a continuous cell culture in the medium for 24 h. Wounded areas were captured at 0 and 24 h using a digital camera system. Representative images of migrating cells were captured via microscopy at 40× magnification. Cell mobility was estimated using the ratio of migration distance, which was evaluated using the software program Celleste 5 (Thermo Fisher Scientific).

### 4.9. Chemoresistance Test

Cells were seeded into 96-well plates at more than 90% confluence for 72 h following chemotherapy. Treatment with oxaliplatin and 5-fluorouracil was performed on the following day. After 72 h, cell viability was evaluated via MTS assay (Promega Corporation), according to the manufacturer’s guidelines. A total of 20 μL MTS solution was added to each well, followed by cell conditioning in a 5% CO_2_ incubator at 37 °C for 2 h. The absorbance of each well was evaluated at a wavelength of 490 nm using an ELISA plate reader.

### 4.10. Phosphoproteomics Database Analysis

The bioinformatics tool Quokka web server was used to identify site-specific kinases that phosphorylate SFRP4 at T186 and T189. Putative kinases with scores greater than 0.5 were selected (http://quokka.erc.monash.edu/, accessed on 24 July 2020).

### 4.11. Confocal Microscopy

For visualization of β-catenin, cells were plated at a cell density of 5 × 10^4^ in a 35 mm confocal dish and treated with Wnt3A-conditioned media. The cells were fixed and permeabilized with methanol for 10 min. The cells were blocked with PBS + 5% bovine serum albumin (BSA). The cells were incubated overnight at 4 °C with anti-β-catenin mouse at a 1:200 dilution in the PBS + 2% BSA. After primary serum incubation, the cells were washed three times with PBS and incubated for 1 h with goat anti-mouse IgG Alexa Flour 568 (Invitrogen) at 1:400 dilution at room temperature. Finally, the cells were washed five times with PBS, and then DAPI mounting solution was mounted to stain the nucleus. The images were captured by using a confocal microscope (LSM-700, Carl Zeiss, Oberkochen, Germany) and ZEN software (Blue edition, version 1.1.2.0; Carl Zeiss, Oberkochen, Germany), which was designed for the acquisition and processing of confocal images.

### 4.12. UALCAN and Kaplan–Meier Plot Analysis

SFRP4 expression in patients with gastric cancer pathology was analyzed using UALCAN (http://ualcan.path.uab.edu/, accessed on 8 December 2022) and the KM plot database (https://kmplot.com/, accessed on 10 March 2022). TCGA data were used in the UALCAN analysis. Overall survival, first progression, and post-progression survival rates were analyzed using the KM plot database.

### 4.13. Statistical Analysis

The data represent the mean ± standard deviation obtained from three independent experiments. Statistical significance was determined via Student’s *t*-test and two-way ANOVA, which was performed using Prism5 (GraphPad Software, La Jolla, CA, USA). *p* < 0.05 was considered for statistical significance.

## Data Availability

The data presented in this study are available upon request from the corresponding author.

## Supplementary Materials

The following supporting information can be downloaded at https://www.mdpi.com/article/10.3390/ijms26125572/s1.

## Abbreviations

The following abbreviations are used in this manuscript:

5-FU	5-fluorouracil
ACRG	Asian Cancer Research Group
CM	Conditioned media
DKK1	Dickkopf-1
GC	Gastric cancer
HCC	Hepatocellular carcinoma
IC_50_	Half maximal inhibitory concentration
PKA	Protein kinase A
p-PKA	Phosphorylated PKA
p-Ser/Thr	Phosphorylated serine/threonine
p-β-catenin	Phosphorylated β-catenin
SEM	Stem-like/EMT/Mesenchymal
SFRP4	Secreted Frizzled-related protein 4
STAD	Stomach adenocarcinoma
TCGA	The Cancer Genome Atlas
YCC	Yonsei Cancer Center
