# Supplementary material for: PKA-Mediated Phosphorylation of SFRP4 Promotes Wnt/β-Catenin Activation and Cancer Stemness in Gastric Cancer
Source: Int J Mol Sci. 2025 Jun 11;26(12):5572. doi: 10.3390/ijms26125572 (PMC12192866; doi:10.3390/ijms26125572)

Figure S1. High SFRP4 expressing gastric cancer cell lines are chemoresistant. (A) Endogenous expression of SFRP4 was profiled in 27 gastric cancer cell lines grouped by growth properties. (B) Cell viability assay shows the average cell viability of gastric cancer cells. Seven gastric cancer cells were treated with 5-fluorouracil for 72 h.

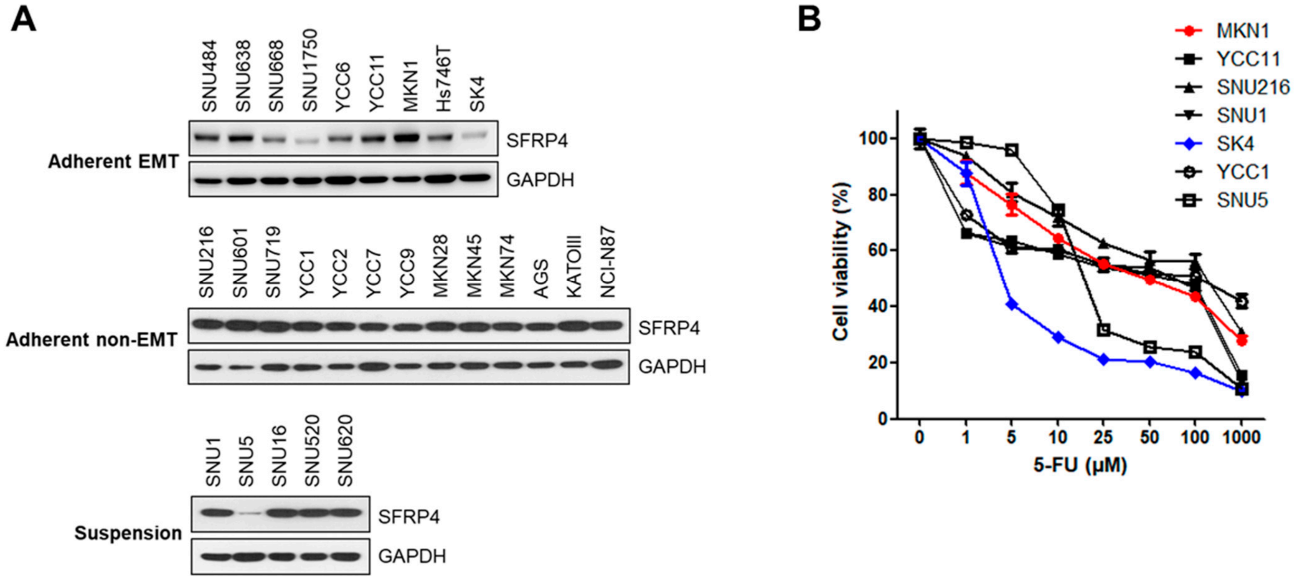

Figure S2. The effect of overexpression and knockdown of SFRP4 on Wnt activity. (A) Overexpression of SFRP4 in SFRP4-low SK4 was confirmed through Western blot (left) and qRT-PCR (right). (B) Knockdown effect of SFRP4 targeted siRNAs in SFRP4-high MKN1 was confirmed through Western blot (left) and qRT-PCR (right). (C) Wnt activity was measured via dual-luciferase system following transfection of pCMV–SFRP4 into SK4 to overexpress SFRP4 and shSFRP4 into MKN1 to knockdown SFRP4. The data of (A–C) are presented as the mean ± standard deviation (n = 3), and p-values were assessed using a two-tailed Student's t-test. * $p < 0.05$, ** $p < 0.01$, *** $p < 0.001$.

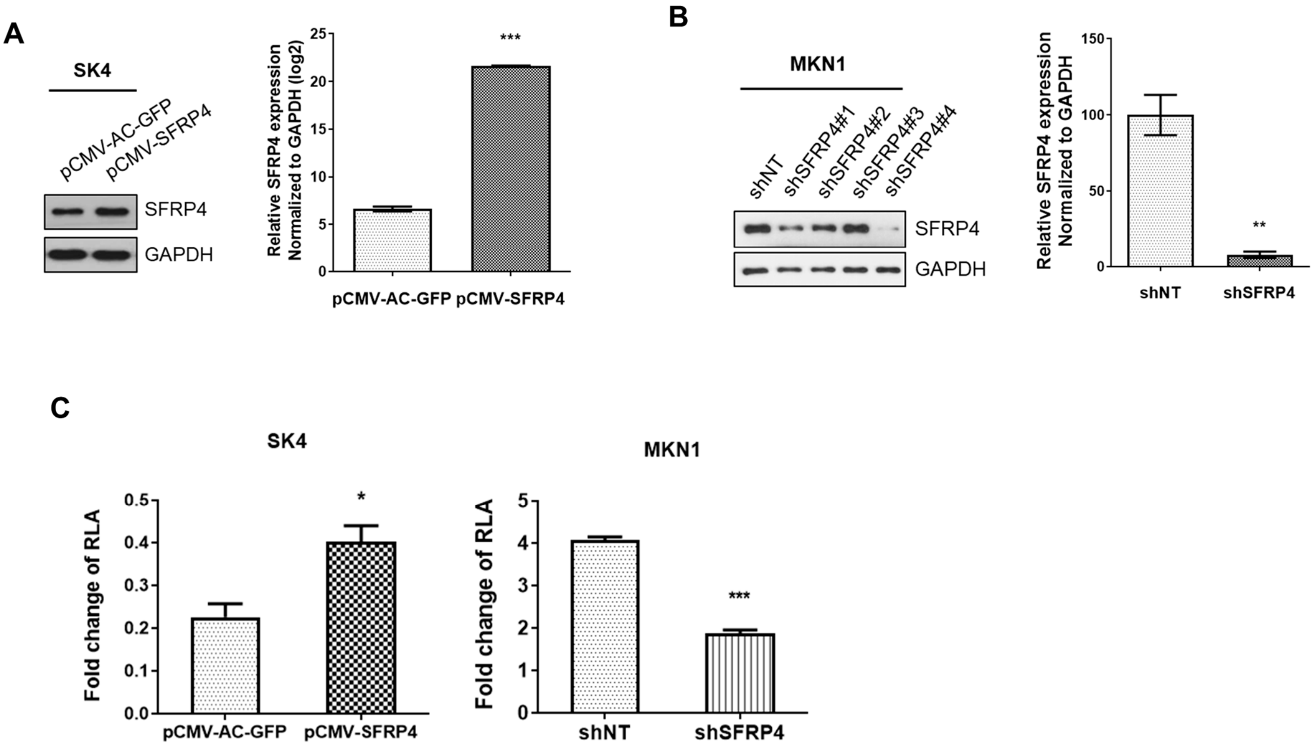

Figure S3. Pharmacological inhibition of PKA through H-89 treatment. (A) Phosphorylated serine/threonine (p-Ser/Thr) was detected through Western blot after treatment with 10 μM H-89 and Wnt3A-conditioned media for 24 and 16 hours, respectively. (B) Western blot was performed to assess the level of β-catenin and SFRP4 after treatment with 10 μM H-89 and Wnt3A-conditioned media for 24 and 16 hours, respectively.

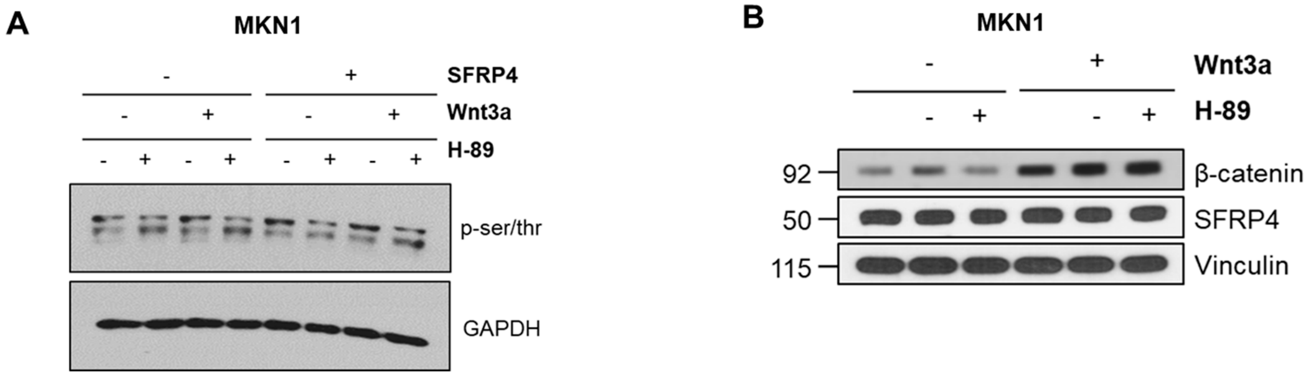

Supplement: Supplementary file 1 [file ijms-26-05572-s001.zip › ijms-3631912-supplementary-english.pdf]
